# Cognitive Impairment Affects Physical Recovery of Patients with Heart Failure Undergoing Intensive Cardiac Rehabilitation

**DOI:** 10.1155/2012/218928

**Published:** 2012-12-30

**Authors:** Giuseppe Caminiti, Francesca Ranghi, Sara De Benedetti, Daniela Battaglia, Arianna Arisi, Alessio Franchini, Fabiana Facchini, Veronica Cioffi, Maurizio Volterrani

**Affiliations:** Cardiovascular Research Unit, Department of Medical Sciences, IRCCS San Raffaele, Via della Pisana 235, 00163 Rome, Italy

## Abstract

*Purpose*. To determine whether the presence of cognitive impairment (CI) affects physical recovery of patients with chronic heart failure (CHF) undergoing a cardiac rehabilitation program (CRP). *Methods*. We enrolled 80 CHF patients (M/F = 53/27). CI was evaluated by means of the Mini-Mental State Examination (MMSE), exercise tolerance was evaluated by six-minute walking test (6 mwt). All patients underwent a 6-week CRP program at 50–70% of maximal *V*_O2_. Patients were divided into two groups according to their MMSE (group 1: 16–23; group 2: 24–30). *Results*. MMSE resulted directly related to ejection fraction (*r* = 0.42; *P* = 0.03), and it was inversely related to creatinine (*r* = −0.36; *P* = 0.04). At 6 week group 1 had a lower increase in distance walked at 6 MWT than group 2 (*P* = 0.008). At multivariate logistic regression MMSE 16–23 predicted a reduced exercise recovery in the overall population (OR = 1.84; 95% CI = 1.50–2.18) and in women (OR = 1.42; 95% CI = 1.22–1.75), while it was not predicted in males. *Conclusions*. CI is a marker of advanced CHF and is an independent predictor of lower exercise recovery after CRP.

## 1. Introduction

Lack of physical fitness is a strong predictor of poor prognosis in patients with chronic heart failure (CHF). Conversely exercise training, often performed in the contest of a cardiac rehabilitation program (CRP), is established as adjuvant therapy for these patients [1]. However, not all CHF subjects have the same benefits from CRP. It has been demonstrated that lack of significant improvement in exercise capacity after a CRP worsens the prognosis of CHF independently of other already known predictive factors [2]. To identify factors related to a lower response to CRP program is of remarkable interest for physicians in order to optimize the rehabilitative intervention. However, reasons for an unfavourable response to CRP are still partially known [3].

Cognitive impairment (CI) has been reported in patients who suffer from a variety of cardiovascular disorders [4] and it has been recognized to affect over one-third of older patients with CHF [5]. Several factors contribute to the high prevalence of CI among CHF subjects, the most important being the cerebral hypoperfusion due to a reduced cardiac output [6, 7]. Conversely CHF is associated with changes in brain regions that are important for demanding cognitive processing [8]. From a clinical point of view, the presence of CI in CHF patients has a negative impact on the course of the disease. At first CI may negatively influence self-management of CHF by reduced medication adherence and their failure to recognize early symptoms awareness. Moreover, CI seems to be related to a more complex clinical profile of CHF patients and to a worst outcome [9, 10]. 

Recently it has been hypothesized that CI could be also related to lack of improvement with exercise training in patients undergoing CRP [11]. 

The prevalence of CI among patients with CHF undergoing CRP and its impact on exercise recovery have not well investigated. Because of its potential deleterious effects on CHF management we postulated that CI could determine lack of response to exercise training performed by CHF patients in the context of CRP. 

The aim of the study was to determine whether the presence of CI affects physical recovery of patients with CHF undergoing a cardiac rehabilitation program after a recent episode of acute decompensation.

## 2. Methods

We evaluated 126 consecutive patients admitted to Cardiac Rehabilitation Unit of IRCCS San Raffaele in Rome evaluated for undergoing an aerobic cardiac rehabilitation program as in-hospital patients, between May 2009 and November 2010. Inclusion criteria were left ventricular ejection fraction (LVEF) < 40%, history of CHF (at least 6 months), and a recent cardiac decompensation (<2 months). Exclusion criteria were inability to exercise, history of claudicatio, clinical instability, ventricular arrhythmias, primary valve disease, congenital heart disease, hypertrophic or restrictive cardiomyopathy, acute coronary syndrome within the past 2 weeks, or patients with planned coronary revascularization or cardiac surgery, active myocarditis, severe COPD, and pericardial effusion. Moreover, in order to exclude subjects with more advanced CI or dementia patients with MMSE score < 16/30 were not admitted to the study.

At entry baseline, anthropometric, clinical, morphological, and biochemical variables were collected by the medical and nonmedical staff. In the first 48 h after admission all eligible patients underwent fasting blood sample collection, evaluation of cognitive function though the administration of the Mini-Mental State Evaluation (MMSE). Glomerular filtration rate was calculated through the Modification of Diet in Renal Disease (MDRD) formula [12]. Exercise tolerance was evaluated by the six-Minute Walking Test (6 MWT). Patients enrolled were divided in two groups according to the MMSE score obtained; group 1 with MMSE between 16 and 23 (43 patients); group 2 with MMSE between 24 and 30 (37 patients). Then all patients underwent an intensive 6-week program of aerobic CRP.

**Assessment of Exercise Tolerance**

Exercise tolerance was evaluated by 6 MWT that was performed at admission and before discharge. The test was performed according to the standardized procedure [13] and was supervised by a physical therapist. Patients were asked to walk at their own maximal pace a 100 m long hospital corridor with 10-meter signs on the floor. Every minute a standard phrase of encouragement was told. Patients were allowed to stop if signs or symptoms of significant distress occurred (dyspnoea, angina), though they were instructed to resume walking as soon as possible. Results of 6 MWT were expressed in distance walked (metres). Functional recovery was defined as the increase of the distance walked at 6 MWT performed at the end of CRP with respect to the 6 MWT performed at baseline (Δ6 MWT).

**Assessment of Cognitive Impairment**

CI was investigated through the Mini-Mental State Examination (MMSE) [14], which is the most commonly administered psychometric screening assessment of cognitive functioning. The MMSE scale ranges from 0 to 30 and includes 10 domain items, which measure orientation to time, orientation to place, registration, attention and calculation, recall, naming and repetition, comprehension, reading ability, writing ability, and design copy.

According to the literature published on the MMSE, it is a relatively sensitive marker of CI [15]. MMSE was administered at admission by a trained psychologist.

**Physical Rehabilitation Program**

It was performed according to the AHA guidelines [16]. Every exercise session included warm-up, cooling down and flexibility exercises, and 30–60 minutes of submaximal aerobic exercise with cycling or treadmill at 50–70% of their maximal theoretical *V*_O_2__. Patients underwent two exercise sessions every day for 6 days/week over a six-week period.

### 2.1. Statistical Analysis

Results are expressed as median ± standard deviation (SD) or percentages where appropriate. Baseline characteristics of patients with and without CI were compared with *t*-tests for continuous variables that were normally distributed, Wilcoxon Mann-Whitney test for continuous variables that were not normally distributed, and Chi-square tests for dichotomous variables. Relations between variables were assessed by Pearson correlation or Spearman's rank test for nonnormally distributed data. Prediction power of CI on functional recovery was evaluated through logistic regression analysis. The difference of distance walked at 6 MWT between baseline and the the end of the CRP (Δ6 MWT) was dichotomized according to its median value (105.3 m). We considered as having a good exercise recovery subjects with Δ6 MWT over 105.3 meters and weak exercise recovery those with Δ6 MWT below 105.3 meters. This categorical variable was used as dependent variable in the logistic regression analysis. The regression model was applied to the overall population, to the male and female genders. A value of *P* < 0.05 was considered significant. All analyses were performed using a commercially available statistical package (SPSS for Windows 12.0, Chicago, IL, USA).

## 3. Result

Out of 126 patients screened 85 patients (age 72.6 ± 10.6; M/F 53/27) met the inclusion criteria. Forty-one patients were not included because they were not able to perform a 6 MWT at admission because of their poor clinical conditions. Another five patients out of 85 were not included in the study. Two patients (2.3%) died during the hospitalization for acute decompensation, before starting CRP, and both had MMSE 16–23. Three patients with MMSE 16–23 and one with MMSE 24–30 were not included because they did not start the CRP; the reason for noninclusion was in every case worsening clinical status needed management in acute care. 80 patients completed the CRP and final evaluations and their data were considered for the study.

MMSE 16–23 was found in 54.4% of the overall population. 29 (55.2%) male and 14 (52.7%) female patients had MMSE 16–23. Overall females had a lower MMSE score than males (22.3 ± 4 versus 24.9 ± 5, *P* = 0.07).

Baseline clinical features of our patient population are reported in Table 1. Patients of group 1 were older, had an higher resting heart rate, and had more often diabetes and atrial fibrillation than patients of group 2. Moreover, patients of group 1 had a lower LVEF and higher levels of NT-pro-BNP and NYHA class than patients of group 2.

The score obtained at MMSE resulted directly related to EF (*r* = 0.42; *P* = 0.03) and it was inversely related to creatinine levels (*r* = −0.36;  *P* = 0.04).

At the end of the study the distance walked at 6 MWT increased in both groups compared to baseline (group 1: from 155.4 ± 36 m to 253.1 ± 47 m; group 2 from 182.6 ± 42 m to 313.7 ± 69 m) with a significant greater increase in group 1 compared to group 2 (*P* = 0.004). Among patients of group 1 males had a greater increase of distance walked at 6 MWT compared to females (males: from 168.4 ± 56 m to 275.5 ± 45 m; females: from 131.7 ± 26 m to 213.1 ± 34 m; between-gender *P* = 0.03). In group 2 males and females had a similar increase of distance walked at 6 MWT (males: from 208.4 ± 49 m to 360.8 ± 61 m; females: from 178.4 ± 57 m to 284.3 ± 61 m; between-gender *P* = 0.09).

The independent prediction power of CI on functional recovery was evaluated through a logistic regression analysis in which we included as covariates some confounding variables such as diabetes, creatinine, haemoglobin, age, ejection fraction, and atrial fibrillation (Table 2). After adjusting for these covariates the presence of lower CI resulted significantly is related to a lower functional recovery in the overall population (adjusted OR = 1.84; 95% CI = 1.50–2.18, *P* = 0.024). Repeating the regression in each gender analysis and adjusting for the same covariates, CI maintained its significant predictor power only in the female gender (OR = 1.42; 95% CI = 1.22–1.75, *P* = 0.047), while there was not independent association between CI and functional recovery among males.

## 4. Discussion

The present study shows three important findings. First, CI has a high prevalence rate among patients with CHF undergoing a cardiac rehabilitation program after an acute cardiac event. Second, our data suggest that the presence of CI could be a marker of clinical complexity of patients and of a more advanced stage of CHF. Third, CI in these patients is associated with a reduced response to the exercise training program. 

In our cohort 54% of CHF subjects had a MMSE 16–23 corresponding to a moderate-to-severe CI. There is a great variability in the literature concerning the prevalence of CI among CHF patients. In the review of Almeida and Flicker [5], it ranges from 25% to 74%. This variability observed can probably be explained by diverse study designs, CHF severity, age of patients, sample sizes, instruments used to assess cognitive impairment, and diagnostic criteria between different studies. However, our results are in line with previous studies considering similar population of elderly CHF patients hospitalized or recently discharged from acute care facilities [17, 18]. In the cross-sectional study of Zuccalà et al. [17] on 57 consecutive CHF inpatients with no prior history of dementia, with a mean age of 76.7 years, 53% scored below 24/30 on the MMSE. 

Several factors contribute to the high prevalence of CI among CHF, the most important being the cerebral hypoperfusion due to a reduced cardiac output [3, 4]. This is demonstrated by an increasing body of evidence suggesting that a decreased heart function is independently associated with impairment in various cognitive domains [5, 6]. Alternatively, a multiple-cardiogenic emboli hypothesis has been advanced and this hypothesis is in agreement with the higher rate of atrial fibrillation among patients of group 1 we observed. 

In our study patients with CHF and CI had a more severe clinical profile with a higher number of comorbidities than patients without CI. Patients of group 1 were older, with an higher resting heart rate, a higher rate of diabetes, and atrial fibrillation than patients of group 2. Moreover, group 1 had a more advanced stage of heart disease as demonstrated by the higher NYHA stage, lover LVEF, and higher levels of NT-proBNP. Our observations are widely confirmed in the literature; Zuccalà et al. [18] demonstrated that CI among patients with CHF is associated with several comorbid conditions, some of which are potentially treatable. Moreover, a strong association between the severity of CI from a side to the severity of LV systolic dysfunction and severity of NYHA symptoms from the other side has been observed by other authors [19, 20]. Taken together these data seem to indicate that CI is a marker, easy to assess, and of clinical complexity of CHF patients who adhere to a CRP after an acute clinical event. Our data are also in agreement with other studies demonstrating that CI has an adverse impact on disease course by influencing the burden of disease, survival rates, and resource consumption. In a study investigating the in-hospital mortality among CHF patients, CI was found to increase the mortality by five times [9]. Recently O'Donnel et al. [21] found in a large population of patients with prior cardiovascular disease an inverse association between baseline MMSE score and risk of stroke, hospitalization for CHF, and death. That association was independent of all other prognostic factors. In a 5-year follow-up study, McLennan et al. [22] found that even patients who had mildly impaired cognition at baseline experienced significantly reduced event-free survival and overall life expectancy. 

Comparing the results of the two 6 MWTs performed before and after CRP, we observed a significant improvement in the distance walked for both group 1 and group 2 subjects. However, group 2 reached an almost complete functional recovery after CR while group 1 subjects seem to have less benefits from CRP. In our cohort an MMSE score 16–23 was predictive of a lower exercise recovery as demonstrated by the multivariate logistical regression analysis also after adjusting for several confounding variables. It is well known that the response to CRP among CHF patients is variable. Lack of improvement of exercise capacity after training may be due to clinical parameters related to heart failure or to several other factors such as intercurrent illness, exacerbation of disease (e.g., acute coronary syndrome), injury (e.g., orthopedic complications), inadequate adherence to the exercise prescription, and poor patient compliance. The possibility to predict a poor response to CRP appears as a relevant information for physicians who plan the rehabilitative intervention. Tabet et al. [2] have shown that the absence of improvement in exercise capacity after an exercise training programme is a predictor of poor prognosis in patients with CHF. The concept that patients with CI may experience reduced benefit from an exercise-based cardiac rehabilitation program has been recently proposed. Kakos et al. [23] analyzed a cohort of forty-four older adults enrolled in a 12-week exercise-based CRP. Cognitive function was investigated through the Trail Making Test Part B, a measure of executive functions, and MMSE. Authors demonstrated that patients with poorer executive functions at baseline derived less benefit from their course of rehabilitative treatment. Our results suggest that the exercise recovery is reduced mostly in female patients. In our study male patients with CI showed a similar improvement of functional capacity than those without CI, while women with CI had the poorest functional recovery. There are no data for gender differences on the relation between CI and exercise capacity and the underlying mechanisms remain unclear [11]. We speculate that it could depend on a higher degree of CI among females in our cohort as demonstrated by the lower MMSE score they obtained at baseline evaluation. However, because of the limited sample size this data needs further confirmation in larger studies.

**Limitations**

The most important limitation of this study is the small sample size and our data, especially with regard to gender differences, need further confirmation in larger studies. In order to asses CI in this study we used the MMSE. Brief screening instruments such as the MMSE can be easily administered by health professionals in the clinical practice to confirm the presence of cognitive impairment. MMSE, however, may be insufficient in identifying subtle cognitive deficits and more detailed neuropsychological assessment is required. The real number of patients with CI in this study, particularly mild CI, could be underestimated due to the low sensibility of the MMSE for this less severe condition as reported by other authors [24]. During the study, we measured cognitive function only once and we do not know whether cognitive function improved or declined at the end of the study after CRP. At least in this study informations on instrumental social support were not taken into account and this could affect our results and limit our conclusions.

In conclusion, CI is a marker of advanced CHF and reduced physical performance. CHF patients with CI have lower exercise recovery than patients without CI after CRP. Because it is an independent correlation with lower exercise recovery, we suggest that CI should be investigated in every patients with CHF undergoing a cardiac rehabilitation program. The assessment of CI at the admission, together with other baseline evaluations, could help physicians in order to determine patients' risk profile, to predict the response to CRP, and planning an individual tailored rehabilitative intervention. 

## Figures and Tables

**Table 1:** Statistical comparison among baseline variables of subjects of group 1 and group 2.

	Group 1	Group 2
	(MMSE 16–23)	(MMSE 24–30)
	*N* = 43	*N* = 37
Age, y	73.5 ± 13	70.7 ± 11*
M/F	29/14	24/13
BMI (kg/m^2^)	27 ± 8	26 ± 4
NYHA class	2.7 ± 0.5	2.2 ± 0.4*
Resting HR, bpm	88 ± 7	76 ± 7*
Systolic BP, mmHg	108 ± 19	107 ± 21
Diastolic BP, mmHg	82 ± 10	80 ± 14
Echography		
LVEF	27.4 ± 7	34.9 ± 6*
LVDD	63.1 ± 11	62.6 ± 8
E/A	1.4 ± 0.7	1.4 ± 0.8
E deceleration time	171 ± 23	180 ± 17
Laboratory tests		
NT proBNP, pg/mL	302.7 ± 51	223.4 ± 34*
Creatinine, mg/dL	1.9 ± 0.3	1.4 ± 0.5*
GFR, mL/min	34.9 ± 9	50.1 ± 7*
Haemoglobin, g/dL	10.4 ± 3	11.1 ± 4
Comorbidities		
Hypertension	29 (67)	24 (65)
Diabetes	21 (48)	12 (32)*
Dislipidemia	17 (39)	15 (40)
COPD	16 (37)	11 (30)
Atrial fibrillation	15 (35)	10 (27)*
Therapy		
Beta-blockers	36 (84)	30 (81)
ACE-i/ARBs	39 (91)	36 (97)
Diuretics	34 (79)	27 (73)
Digoxin	9 (21)	6 (28)

*Intergroup differences *P* < 0.05.

GFR: glomerular filtration rate.

LVDD: left ventricular diastolic diameter.

LVEF: left ventricular ejection fraction.

**Table 2:** Logistic regression analysis evaluating the independent predictor power of CI (MMSE < 24) in the overall population, and according to gender.

MMSE 16–23 versus MMSE 24–30	Odds ratio (95% CI)	*P* value
Overall population		
Unadjusted model	2.21 (1.62–2.55)	<0.001
Adjusted model	1.84 (1.50–2.18)	0.024
Males		
Unadjusted model	1.5 (0.98–1.91)	0.036
Adjusted model	1.20 (0.87–1.52)	0.354
Females		
Unadjusted model	1.76 (1.30–2.07)	0.031
Adjusted model	1.42 (1.22–1.75)	0.047

Adjusted for LV ejection fraction, diabetes, age, atrial fibrillation, creatinine, and haemoglobin.
